# Case report: Investigation of genetic mutations in a case of schistosomus reflexus in a Holstein dairy cattle fetus in Korea

**DOI:** 10.3389/fvets.2023.1238544

**Published:** 2023-08-21

**Authors:** Woncheoul Park, Han-Ha Chai, Dajeong Lim, Changgwon Dang, Jaegu Lee, Jongho Kim, Hogyun Jeong, Taekwon Lee, Ki-Chang Lee, Kyunghyun Lee

**Affiliations:** ^1^Animal Genomics and Bioinformatics Division, National Institute of Animal Science, Rural Development Administration (RDA), Wanju-gun, Republic of Korea; ^2^Animal Genetics and Breeding Division, National Institute of Animal Science, Rural Development Administration (RDA), Cheonan-si, Chungcheongnam-do, Republic of Korea; ^3^Animal Disease Diagnostic Division, Animal and Plant Quarantine Agency, Gimcheon-si, Republic of Korea; ^4^Veterinary Medicine College, Jeongbuk National University, Iksan-si, Republic of Korea

**Keywords:** schistosomus reflexus, Holstein, genetic mutation, vertebrae dysplasia, fetus, SNP-Chip

## Abstract

Schistosomus reflexus (SR) is one of the most common congenital anomalies found in cases of cattle dystocia; this disorder occurs mostly in cattle. Congenital anomalies such as SR are caused by various genetic and environmental factors, but no specific cause has been elucidated for SR. This study reports a case of SR in a Holstein dairy cattle fetus with congenital anomalies in Korea. Grossly, a distinct spine curvature was observed between the thoracic and lumbar vertebrae, accompanied by a consequential malformation from the sacrum to the occipital bone. Furthermore, the thoracic and abdominal organs were exposed. In computed tomography (CT) images, mild and severe kyphoscoliosis was observed in T1~11 and L1~6, respectively. Additionally, vertebral dysplasia was observed in S1~5 and Cd 1~5. To pinpoint the causal genes and mutations, we leveraged a custom 50K Hanwoo SNP-Chip and the Online Mendelian Inheritance in Animals (OMIA) database. As a result, we identified a nonsense mutation in apoptotic protease activating factor 1 (*APAF1*) within HH1 that was associated with a decrease in conception rate and an increase in abortion in Holstein dairy cattle. The genotype of the SR case was A/A, and most of the 1,142 normal Holstein dairy cattle tested as a control group had the genotype G/G. In addition, the A/A genotype did not exist in the control group. Based on the pathological, genetic, and radiological findings, the congenital abnormalities observed were diagnosed as SR.

## Introduction

Schistosomus reflexus (SR) is the most form of common congenital anomaly found in cases of cattle dystocia; it occurs mainly in cattle, but is also common in other domesticated animals. The prevalence rate of this fatal congenital syndrome ranges from as low as 0.01% to as high as 1.3% of dystocia cases (1, 2). SR occurs mainly in bovines and rarely in other species such as sheep, goats, pigs, dogs, cats, donkeys, and sea turtles (*Lepidochelys olivacea*) (3–8). The main morphological symptom of SR is a body malformation falling within the category of celosomy. Specifically, SR is characterized by the presence of exposed abdominal and sometimes thoracic viscera (schistosomus), which causes their submersion in amniotic fluid; moreover, it is also characterized by a marked ventral curvature of the thoracic vertebrae (reflexus), which causes the occipital bone to approach the sacrum, lateral curvature of the body and walls of the chest, pelvic deformity, and an abnormally cystic liver shape. These symptoms result in fetal dystocia (1, 9).

Apparent genetic factors that cause SR have not been identified in bovines, such as SNPs, indels, or CNVs. However, there are reports on genes associated with a syndrome similar to SR in other species. First, four genes have been reported in mice: transforming growth factor beta 2 (*TGFB2*) and beta 3 (*TGFB3*), paired-like homeodomain transcription factor 2 (*PITX2*), and activator protein 2 (*AP2*) (10–12). Second, the thoracoabdominal syndrome (*THAS*) gene on the X chromosome has been reported in humans (13, 14). Very few studies have been conducted on the causes of SR, and especially few have examined the genetic factors. In addition, most SR case reports have reported on the clinical signs and pathological lesions in some species. In this study, in order to fill this gap, we collected customized Hanwoo 50k SNP-Chip data to identify genetic factors and monitored the clinical signs and pathological lesions occurring in a case of SR in a calf of Holstein dairy cattle in Korea.

## Case description

**Figure 1:**
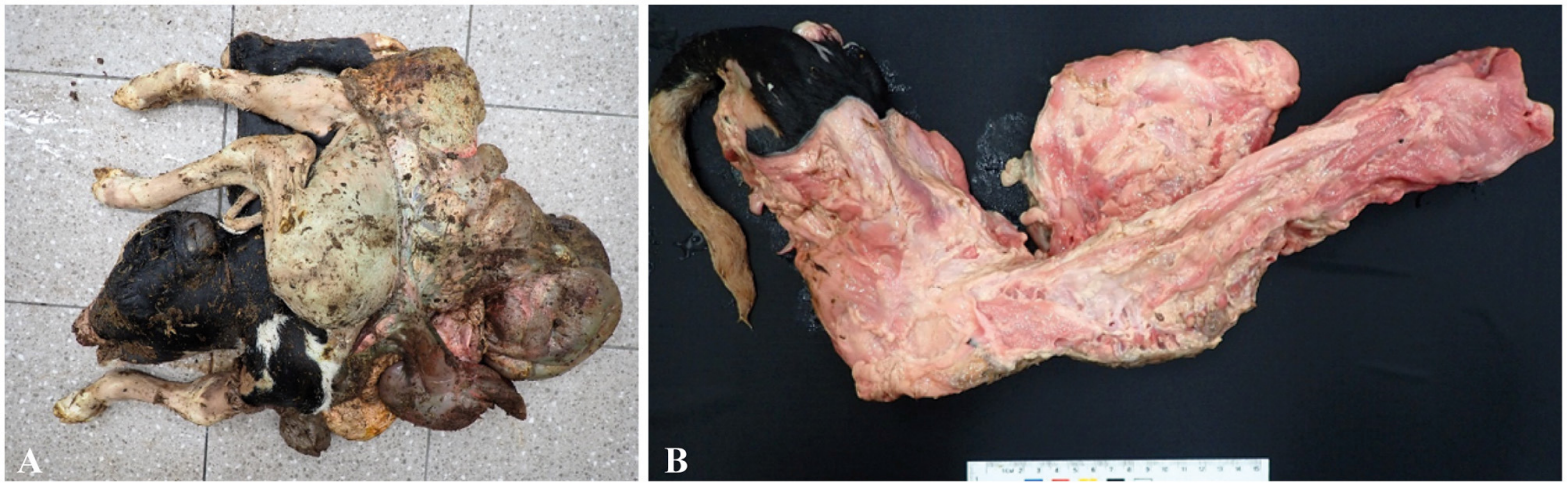
**(A)** Schistosomus reflexus of the fetus: the body and chest walls bent laterally with exposed thoracic and abdominal viscera, with a gross lesion. **(B)** V-shaped bent laterally twisted vertebrae were observed, with a gross lesion.

A Holstein (pure breed) dam had a stillbirth of twins on the 220th day of pregnancy. One fetus had a crown–rump length of 67 cm, a body weight of 14.5 kg, and no specific problems on gross observation. However, the other fetus showed congenital abnormalities. Gross findings indicated full exposure of the thoracoabdominal organs; this observation was followed by additional computed tomography (CT) scanning. Externally, the body exhibited SR with exposed abdominal organs; the spine was curved and inverted laterally and dorsoventrally, and the anterior surface of the posterior limb was directed posteriorly; and the body weighed 11.6 kg (Figure 1A). The thoracic and visceral organs were exposed. The vertebral column bent to the left, and the sacrum approached the cranium, as observed by noting that the caudal lumbar vertebrae presented with a v-shaped lateral twist of the vertebrae (Figure 1B). The diaphragm was intact, and the thoracic cavity was reduced in size. The lung and heart were deformed in shape and size. The liver was markedly deformed in shape and thickness. The pelvic cavity was reduced in size by compression laterally to the left.

**Figure 2:**
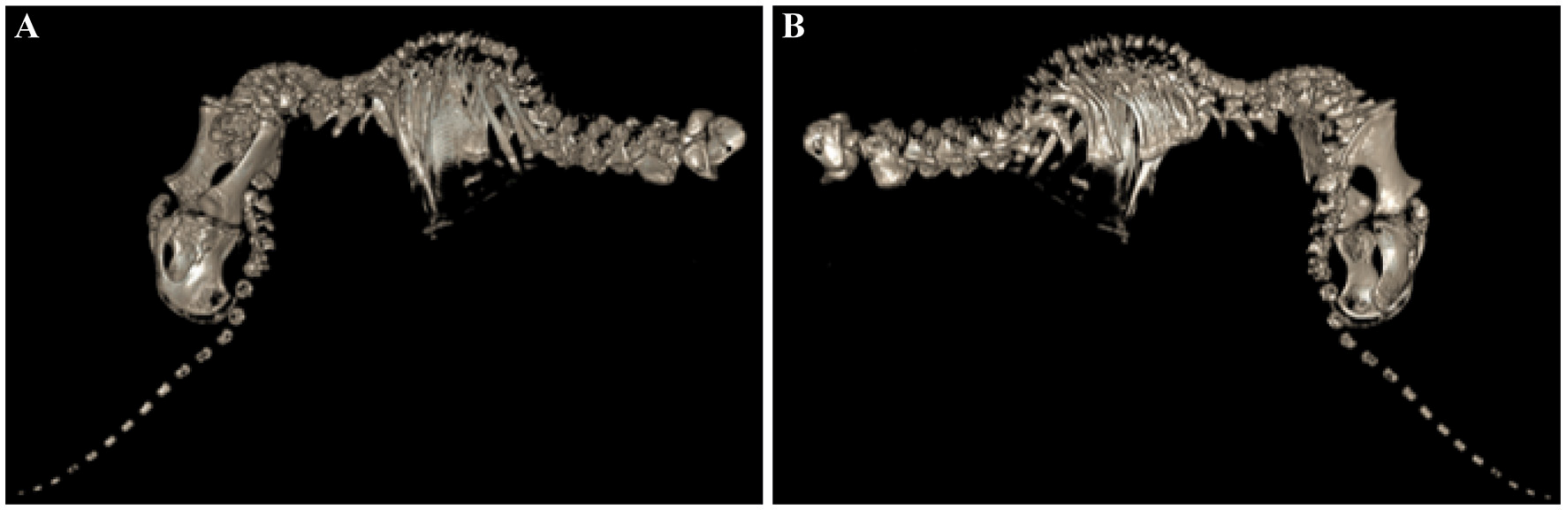
CT image of schistosomus reflexus of the fetus. A developmental defect characterized by a marked curvature of the spine and a deformed pelvis. **(A)** Kyphoscoliosis was mild in T1~11 and severe in L1~6; left view of the figure. **(B)** Vertebral dysplasia was observed in S1~5 and Cd 1~5; right view of the figure.

In the CT images, seven cervical vertebrae and the alignment of these vertebrae were relatively normal. Bone density in the cervical vertebral body was generally decreased, and all the endplates were unfused. There was a 1-mm-wide gap longitudinally along the central aspect of the C1 (atlas) vertebral arch (Figure 2A) and also a 1-mm-wide gap longitudinally along the left lateral and right lateral aspects of the vertebral body. These gaps were assessed to be a non-fused vertebral arch and vertebral body. The C2 vertebra (axis) was observed to be divided into two parts, cranial and caudal. The cranial part of C2 was observed to have a round shape with a thickness of approximately 17 mm within the vertebral foramen of C1. The caudal part of C2 was observed without structures such as the vertebral body and the spinous process. There were only 12 thoracic vertebrae, which is one less than the normal number in this species. Abnormal formation of the vertebral curvature was noted along the dorsal aspect of T1-11. The endplates of these vertebrae were all unfused (Figure 2B). The left 1st to 3rd ribs appeared relatively normal. At the T4-8 level (left), approximately five consecutive ribs were fused, giving an abnormal appearance around this region. At the T10-12 level (left), three distinct ribs were observed, with the distal parts of the 11th and 12th ribs fused (Figure 2A). The 1st rib on the right appeared short and thick. At the T1-3 level (right), approximately three consecutive ribs were fused, giving an abnormal appearance around this region. At the T4-9 level (right), approximately seven consecutive ribs were also abnormally fused.

There were six lumbar vertebrae among the lumbar, sacral, and caudal vertebrae. The vertebral curvature along the dorsal aspect of the L1-5 appeared extremely abnormal, with all the endplates unfused and five sacra with irregular margins of the vertebral body for S1-5. Overall bone density was observed to be decreased. The left sacral wing of S1 was articulated with the left iliac wing, while the right sacral wing was separated from the right iliac wing. There were 15 caudal vertebrae. The vertebral bodies of Cd1-5 had irregular margins. In the forelimb and hindlimb, the left humerus was caudodistally displaced and luxated from the left shoulder joint (Figure 2A). The physis of the long bones of the fore- and hindlimbs was unfused. No other significant findings were noted in the fore- and hindlimbs. The curved formation noted in the thoracic vertebrae and lumbar vertebrae is related to kyphoscoliosis, a congenital dysplasia. In addition, the 2nd cervical vertebra was partially formed, with only the vertebral arch present; bilateral fused ribs and the irregular margins of the sacrum and the caudal vertebrae were all assessed to be hypoplastic abnormalities and congenital dysplasias. The separated right sacroiliac joint may have been attributable to sacral dysplasia causing SI joint luxation. The overall low bone density observed may have been due to decay of the bone post-mortem or abnormal growth of the fetus in the form of bone marrow hypoplasia. The gaps and the physis observed in the vertebrae and the limbs are considered normal in an undeveloped fetus.

### Genetic analysis

Our genotyping process was as follows. First, the muscle tissues of the Holstein dairy cattle calf with SR, which had been stored in a deep freezer (−78°C), were thawed, washed, chopped, and then placed in 600 μl of nuclei lysis solution. Second, total genomic DNA was extracted using a Wizard genomic DNA purification kit (Promega, Madison, WI, USA), following the manufacturer's instructions. Subsequently, the DNA concentration and purity were measured using a NanoDrop 1000 spectrophotometer (Thermo Fisher Scientific, Wilmington, DE, USA). Finally, the genomic DNA samples were genotyped using a custom 50K Hanwoo SNP-Chip (Illumina, South Korea) involving 58,990 SNPs. In addition, we obtained custom 50K Hanwoo SNP-Chip data from 1,142 Holstein dairy cattle from the Animal Breeding & Genetics Division of the National Institute of Animal Science (NIAS); these data were used to confirm the genotype of normal Holstein dairy cattle as a control group for comparison with the SR case.

**Figure 3:**
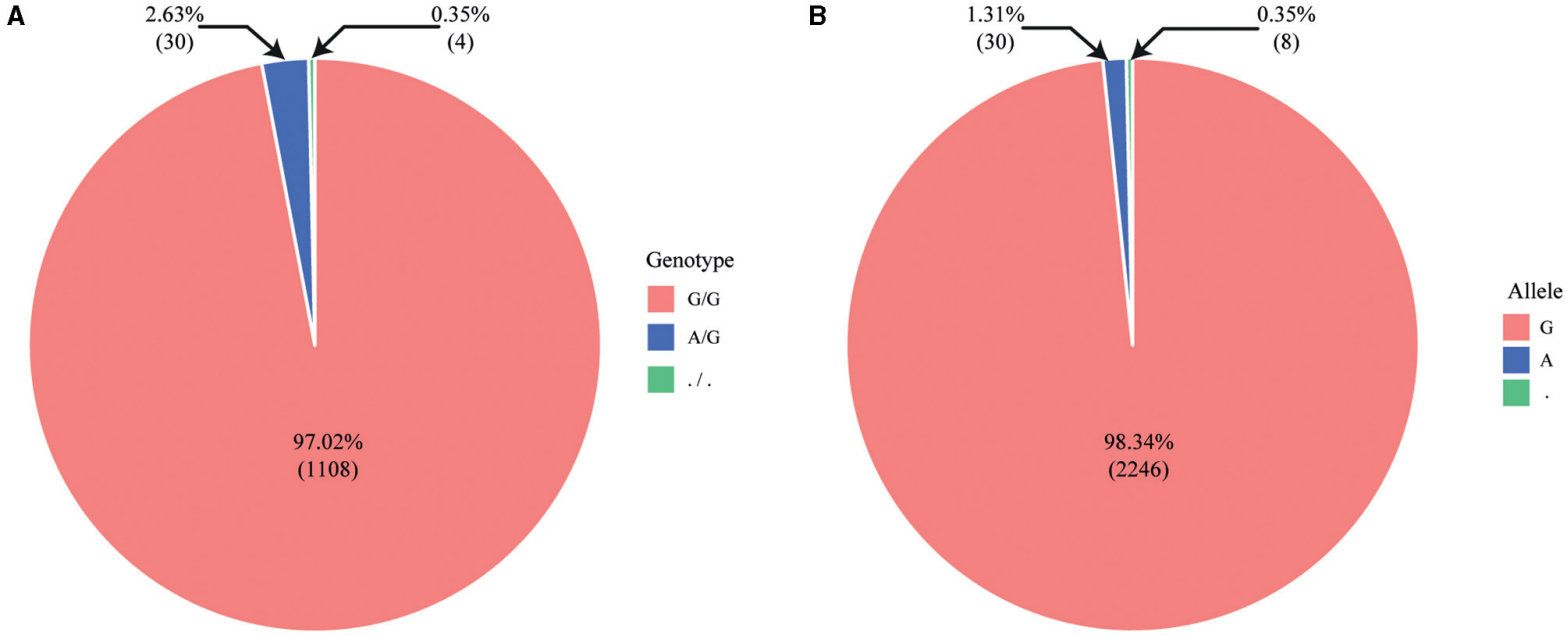
Pie chart illustrating genotype and allele frequency using SNP-Chip data from 1,142 Holstein dairy cattle. **(A)** Genotype frequency (G/G, A/G,./.); **(B)** allele frequency (G, A,.).

We obtained detailed information, including the genotypes for genetic disorders in cattle, from the Online Mendelian Inheritance in Animals (OMIA) database. Subsequently, we summarized the data on 32 genetic disorders that were common between the OMIA database and the custom 50K Hanwoo SNP-Chip data (Table 1). Additionally, we identified the SNP-Chip data for SR in calves of Holstein dairy cattle. As a result, a recessive homozygous genotype (A/A) was identified for abortion due to a haplotype HH1 [Chip ID: ilmnseq_rs448942533-148_B_F_2604252564, apoptosis peptide activating factor 1 (*APAF1*)], which has been reported as a lethal gene for cattle, and dominant homozygous genotypes were identified for other genetic disorders. Moreover, abortion due to the haplotype HH1 genotype was identified as follows among the 1,142 normal Holstein dairy cattle: 1,108 dominant homozygous (normal, G/G), 30 heterozygous (carriers, G/A), and 4 no genotype information (./.). The G and A allele frequencies were 98.33% and 1.31%, respectively (Figure 3). This result confirmed that the A allele frequency of the haplotype HH1 genotype is being reduced through selection in the Holstein dairy cattle bred at the NIAS, and abortion caused by the haplotype HH1 is also being reduced.

**Table 1:** Summary of the custom 50 k Hanwoo SNP-Chip genotype of schistosomus reflexus (SR) calf in Holstein dairy cattle using cattle inherited disorders from the OMIA database.

**No**	**Inherited disease**	**SNP-Chip ID**	**Gene symbol**	**Chr**	**Position**	**DHG^a^**	**HG^b^**	**RHG^c^**	**SR genotype**
1	Abortion due to haplotype HH1	ilmnseq_rs448942533-148_B_F_2604252564	APAF1	5	63150400	GG	GA	AA	AA
2	Abortion due to haplotype HH4	ilmnseq_rs465495560-148_T_F_2604252576	GART	1	1277227	AA	AC	CC	AA
3	Abortion due to haplotype MH1	ilmnseq_rs383770500-148_T_R_2604252509	PFAS	19	28511199	GG	GA	AA	·/·
4	Abortion due to haplotype MH2	SLC37A2_dup-1_B_F_2327736346	SLC37A2	29	28879810	GG	GA	AA	GG
5	Abortion due to haplotype FH4	ilmnseq_rs110793536-148_B_R_2604252459	SUGT1	12	11131497	AA	AG	GG	AA
6	Abortion due to haplotype JH1	CWC15_r2-1_B_F_2277749190	CWC15	15	15707169	GG	GA	AA	·/·
7	Arthrogryposis, distal, type 1B	ilmnseq_g.65787153T>G-148_B_F_2604252380	MYBPC1	5	65787153	AA	AC	CC	AA
8	Arthrogryposis, lethal syndrome	ilmnseq_rs451004237-148_B_R_2604252569	PIGH	10	79814520	GG	GC	CC	GG
9	Citrullinemia	ASS1_dup-1_B_F_2327673206	ASS1	11	100802781	GG	GA	AA	GG
10	Chediak-Higashi syndrome	CHS1_dup-1_T_F_2327728833	LYST	28	8508619	AA	AG	GG	AA
11	Complex vertebral malformation	ilmnseq_rs438228855-148_B_R_2604252559	SLC35A3	3	43412427	CC	CA	AA	CC
12	Congenital muscular dystonia 1	ilmnseq_g.26191380C>T-148_T_R_2604252373	ATP2A1	25	26191380	GG	GA	AA	GG
13	Deficiency of uridine monophosphate synthase	UMPS_dup-1_T_R_2327737250	UMPS	1	69756880	GG	GA	AA	GG
14	Hemophilia A	ilmnseq_rs456129807-148_B_F_2604252570	F8	X	38971744	AA	AC	CC	AA
15	Hypotrichosis	HEPHL1_dup-1_B_R_2327735157	TSR2	29	695072	TT	TA	AA	TT
16	Mannosidosis, beta	ilmnseq_g.23540228G>A-148_T_F_2604252370	MANBA	6	23540228	GG	GA	AA	GG
17	Marfan syndrome	FBN1_1_r3-1_B_R_2277751555	FBN1	10	62054844	GG	GA	AA	GG
18	Male subfertility	TMEM95_r2-1_T_F_2277749282	TMEM95	19	27689622	CC	CA	AA	CC
19	Mucopolysaccharidosis IIIB	ilmnseq_g.43264699G>A-148_B_R_2604252377	NAGLU	19	43264699	GG	GA	AA	GG
20	Muscular hypertrophy (double muscling)	ilmnseq_rs449270213-148_T_R_2604252567	MSTN	2	6213889	AA	AG	GG	AA
21	Myoclonus	GLRA1_r2-1_B_F_2277749211	GLRA1	7	65080197	CC	CA	AA	CC
22	Perinatal weak calf syndrome	IARS_r2-1_T_F_2277749215	IARS.	8	85341291	CC	CG	GG	CC
23	Pseudomyotonia, congenital	ilmnseq_g.26198573G>A-148_T_F_2604252374	ATP2A1	25	26198573	GG	GA	AA	GG
24	Pseudomyotonia	ATP2A1_3_dup-1_T_F_2327673212	ATP2A1	25	26197429	CC	CA	AA	CC
		ATP2A1_2_dup-1_T_F_2327673210		25	26197204	CC	CA	AA	CC
25	Protoporphyria	FECH-1_T_R_2276841199	FECH	24	57298883	GG	GA	AA	GG
26	Ptosis, intellectual disability, retarded growth and mortality (PIRM) syndrome	ilmnseq_rs475678587-148_B_F_2604252582	UBE3B	17	65921497	GG	GA	AA	GG
27	Spherocytosis	SLC4A1_dup-1_T_F_2327736348	SLC4A1	19	44695843	GG	GA	AA	GG
28	Spinal dysmyelination	ilmnseq_rs445770480-148_B_R_2604252563	SPAST	11	14760164	GG	GA	AA	GG
29	Syndactyly (mule foot)	ilmnseq_rs109636878_ilmndup1-148_B_R_2609345037	LRP4	15	77675440	GG	GA	AA	GG
		LRP4_6_dup-1_T_R_2327735627		15	77682052	GG	GA	AA	GG
		LRP4_7_dup-1_T_R_2327735629		15	77686731	GG	GA	AA	GG
30	Tail, crooked	ilmnseq_rs466131011-148_B_F_2604252578	MRC2	19	47734925	AA	AC	CC	AA
31	Trimethylaminuria (fishy taint)	ilmnseq_rs797790546-148_T_R_2604252589	FMO3	16	39523051	GG	GA	AA	GG
32	Zinc deficiency NA-like syndrome	ilmnseq_rs378824791-148_T_F_2604252504	PLD4	21	71001232	GG	GA	AA	GG

a DHG, dominant homozygous genotype.

b HG, heterozygous genotype.

c RHG, recessive homozygous genotype.

## Discussion

Most published works have reported that SR, which occurs mainly in cattle, is diagnosed through necropsy. In addition, studies on SR in various livestock species (bovines, sheep, goats, pigs, dogs, cats, donkeys, turtles, etc.) have been reported. However, studies on ways to prevent the disorder of SR and provide opportune treatment to the pregnant dam have yet to be reported. When SR in a calf is diagnosed early, traction, embryotomy, and cesarean section are used to treat the pregnant dam. However, in cases of delayed diagnosis of SR or decayed emphysematous calves, there are instances in which the dam dies due to poor prognosis. Therefore, to prevent the death of dams, methods for early diagnosis of SR are needed.

HH1, a haplotype on chromosome 5, has been reported to decrease conception rates and increase abortion rates in Holstein dairy cattle; this haplotype was identified by VanRaden et al. through high-density SNP genotyping (15). It has also been reported that the HH1 haplotype originated from a single sire born early in advanced animal breeding over 50 years ago (16). Moreover, this HH1 haplotype carried by the sire was identified as a stop-gain (nonsense) mutation in the *APAF1* gene. The *APAF1* gene is an essential molecule in the cytochrome-c-mediated apoptosis cascade and has been directly implicated in developmental and neurodegenerative disorders. Embryos with these homozygous gene knockouts have been found to die by 16.5 days of development (17).

In this study, we discovered that the HH1 haplotype mutation, which has caused approximately 525,000 spontaneous abortions worldwide over the past 35 years, causing losses of approximately $420 million over the same period (17), is also mutated in the genetic disorder of SR. Previous studies in Holstein dairy cattle have reported diagnoses through pathological methods for calves suspected of having SR. However, based on our results, we recommend diagnosing the genetic disorder of SR in the fetus early on and, at the same time, promptly removing the fetus from the pregnant dam using methods such as traction, embryotomy, and cesarean section. Additionally, more attention should be given to the treatment of the dam. Finally, we believe that, by selecting against the deleterious alleles that cause SR and abortion through systematic breeding schemes, the damage to livestock farms can be reduced by reducing the frequency of carriers in the parental generation.

## Conclusion

This study aimed to identify the pathological symptoms and genetic mutations associated with the genetic disorder of SR, which commonly occurs in cattle. To this end, genetic mutations were identified using SNP-Chip data and pathological symptoms of SR cases in Holstein dairy cattle in Korea. As a result, a recessive allele (A/A) was identified in HH1, previously reported in the OMIA database as a lethal gene that causes abortion in Holstein dairy cattle. If additional SR cases are observed in Korea in thefuture, we will identify genetic mutations in the whole genome using re-sequencing and SNP-Chip data.

## Data availability statement

The original contributions presented in the study are included in the article, further inquiries can be directed to the corresponding author.

## Ethics statement

This study is only field case not the animal experiments with infection. Written informed consent was obtained from the owners for the participation of their animals in this study. Written informed consent was obtained from the participant/patient(s) for the publication of this case report.

## Author contributions

WP and KL designed and performed the research, analyzed the data, and wrote the manuscript. CD and JL provided and analyzed the SNP-Chip data. JK worked on the pathological examination. HJ and TL worked on the chromatography examination and imaging diagnostics. K-CL provided technical comments on the imaging diagnostics. KL handled technical matters for pathology and imaging. H-HC and DL interpreted the results and finalized the manuscript. All authors read and approved the final manuscript.
